# Lifetime and past‐month alcohol use and related factors among female sex workers in Iran

**DOI:** 10.1002/brb3.3288

**Published:** 2023-10-23

**Authors:** Mohammad Aziz Rasouli, Bushra Zareie, Mohammad Mehdi Gouya, Fatemeh Hadavandsiri, Marzieh Mahboobi, Yousef Moradi, Rozhin Moradi, Ghobad Moradi

**Affiliations:** ^1^ Department of Epidemiology and Biostatistics Faculty of Medicine, Kurdistan University of Medical Sciences Sanandaj Iran; ^2^ Department of Epidemiology, School of Public Health Hamadan University of Medical Sciences Hamadan Iran; ^3^ Iranian Center for Communicable Disease Control Ministry of Health and Medical Education Tehran Iran; ^4^ School of Public Health and Saftey Shahid Beheshti University of Medical Sciences Tehran Iran; ^5^ Social Determinants of Health Research Center, Research Institute for Health Development Kurdistan University of Medical Sciences Sanandaj Iran

**Keywords:** alcohol, female sex workers, high risk behaviors, Iran

## Abstract

**Objective:**

Alcohol use is more common among female sex workers (FSWs). This study assessed the prevalence of lifetime and past‐month alcohol use and related factors among FSWs in Iran.

**Methods:**

We conducted a cross‐sectional survey among 1464 women from 8 major cities in Iran. Behavioral data were collected by trained interviewers and conducted face‐to‐face in a private room. Weighted analysis was used to determine the lifetime and past‐month alcohol use prevalence. Univariate and multivariate logistic regression was used to investigate the association between alcohol use and independent variables.

**Results:**

The most alcohol used in lifetime and past‐month (weekly, less than once a week, and daily) in FSWs was 52.7% (12.25%, 12.94%, and 1.83%), respectively. In the final model, factors that were independently associated with alcohol use included the 31–40 years (AOR = 2.41, 95% CI: 1.13–5.15), education level of diploma (AOR = 2.43, 95% CI: 1.31–4.51), history of lifetime drug use (AOR = 2.79, 95% CI: 2.01–3.89), history of lifetime group sex (AOR = 2.07, 95% CI: 1.41–3.03), history of intentional abortion (AOR = 1.42, 95% CI: 1.06–1.92), six or more sexual clients in the last month (AOR = 3.25, 95% CI: 1.80–5.87), history of lifetime anal sex (AOR = 2.47, 95% CI: 1.82‐3.35), and FSWs the married, temporarily married, and living with partner were positively associated with lifetime alcohol use.

**Conclusion:**

Alcohol use is prevalent among FSWs in Iran. Further prevention programs are needed to address and reduce harms associated with alcohol use among this vulnerable population in Iran. Designing intervention programs, it is suggested to consider other variables affecting alcohol use in FSWs.

## Graphical abstract

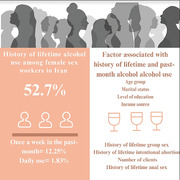

## 1 INTRODUCTION

Female sex workers (FSWs) are defined as women receiving money or gifts in exchange for consensual sexual services or erotic performances, either regularly or occasionally, and because of having multiple partners and sexual contacts, they are vulnerable to sexual health risks (Arinaitwe et al., 2022; Lafort et al., 2016). High rates of alcohol use among FSWs have been reported in low‐ to middle‐income countries around the world, and alcohol use has been significantly higher in these individuals than the general population (Beksinska et al., 2023). A range of adverse health and social consequences related to alcohol use has also been recorded in FSWs. These consequences include an increased risk of STIs, poor mental health outcomes, physical health problems, social stigmatization, violence, and victimization (Beksinska et al., 2023; Rock et al., 2022).

Although women typically alcohol use less than men, the prevalence of alcohol use among FSWs in Iran is indeed higher than that of the general female population (Khezri et al., 2022). In Iran, sex work is illegal and criminalized, and sensitivities around FSWs in Iran are mainly rooted in the sociocultural, religious, and political context of Iran as a Muslim majority setting (Karamouzian et al., 2016; Karamouzian et al., 2020). Although there are limited data on alcohol use, as well as the factors that influence and the outcomes associated with its consumption among FSWs in Iran, studies conducted in the country have revealed that more than 50% of FSWs acknowledge the use of alcohol (Karamouzian et al., 2017; Mousavi‐Ramezanzade et al., 2020).

FSWs may be at risks for the negative health effects of alcohol use. On the one hand, alcohol use is closely related to the number of unprotected sex and can lead to unprotected sex between sex worker and their clients (Amogne et al., 2021; Mann et al., 2005). In addition to individual‐level factors, the increased risk of HIV/STIs among FSWs is further exacerbated by structural risk factors. These factors include poverty, stigma, discrimination, violence, and the criminalization of sex work, all of which create barriers for FSWs in accessing HIV/STIs prevention, care, and treatment services (Karamouzian et al., 2020; Shannon et al., 2015). Therefore, identifying factors related to alcohol use patterns can serve as a guide to reducing alcohol use and mitigating the damages and social consequences associated with it (Karamouzian et al., 2020; Shannon et al., 2015). These consequences include an increased risk of STIs, poor mental health outcomes, physical health problems, social stigmatization, and violence and victimization, which have also been observed among FSWs (Rehm et al., 2009; Shannon et al., 2015). This underscores the significance of implementing strategies to reduce alcohol use and its associated harms. By comprehending the distinctive challenges that FSWs face and addressing the underlying factors that contribute to their alcohol use, it is feasible to enhance the health and well‐being of this group (Beksinska et al., 2023; Lichtwarck et al., 2022).

Although previous research on alcohol‐related factors among FSWs has mainly focused on individual characteristics (such as age and education), other studies have demonstrated that work context and demographic factors are also important predictors of alcohol use (Karamouzian et al., 2017; Mousavi‐Ramezanzade et al., 2020). To mitigate the effects and harms associated with alcohol use among FSWs, it is essential to thoroughly examine the various factors related to alcohol use during and prior to sexual encounters within this population. By conducting comprehensive investigations, we can gain a better understanding of the specific dynamics and risks involved. This knowledge can then inform targeted interventions and strategies aimed at reducing the negative consequences of alcohol consumption in FSWs. We describe the prevalence of alcohol use and associated risk factors among FSWs populations from Iran.

## 2 METHODS

### 2.1 Study design, setting, and population

In this cross‐sectional study, the third round of bio‐behavioral surveillance data in the group of FSWs, called integrated bio‐behavioral surveillance‐III (IBBS‐III), was performed in December 2019 and August 2020. RDS has a long history of use in studies involving FSWs, and the data collected through RDS‐based IBBS‐III are typically highly valuable. RDS is chosen for this group because FSWs are considered hard‐to‐reach, and when it comes to estimating and generalizing findings for such groups, various methods exist. However, RDS is widely recognized as one of the most famous and commonly used methods for studying FSWs (Bernier et al., 2020; Damacena et al., 2019; Hosseini‐Hooshyar et al., 2022). As this study aimed to use the RDS method for the first time, it was not feasible to include a large number of cities. Therefore, the selection of cities was done in a way that would provide a suitable sample representing the entire country. Based on these criteria, the following eight cities were chosen for sample selection: Sari, Tabriz, Tehran, Bandar Abbas, Shiraz, Mashhad, Kermanshah, and Khorram Abad.

According to the formula based on the study (Izadi et al., 2023), the recommended range for selecting samples in each city is a minimum of 150 and a maximum of 250. FSWs define in this study was “a woman who has had sexual contact (vaginal, anal, or oral) with a male customer in exchange for receiving money, drugs, or any other service (including food, phone charging, a place to live, travel tickets, etc.).” The inclusion criteria for FSWs in this study included being born female, at least 16 years old, having sexual contact with several male clients for money in the past year, living or working in the target city for at least 12 months before the interview, and having a valid RDS voucher (excluding seeds) and providing consent to participate in the research.

### 2.2 Seed selection and recruitment

The selection of seeds for the RDS process took into account various factors, including age, geographic location, and risk characteristics associated with specific subgroups. Ultimately, a total of 45 seeds were chosen, with each city having a minimum of 4 seeds and a maximum of 9 seeds. However, one seed did not recruit any participants for the study and was therefore excluded, resulting in a total of 44 seeds available for analysis. It is important to mention that the latest recommendations for the RDS method suggest that the number of seeds should not be less than 5.

Previous studies of FSWs required a qualitative study to know what type of people they are, where they are located, and how they can be accessed. Based on this qualitative information, it was tried that different points that may be selected for this seeds should be based on this information. To conduct the study using the RDS method, the study was first started according to the specified sample size for each city and by selecting a small number of initial participants called “seed.” They were then asked to chain other people of their peers who were eligible to join the study using referral coupons.

### 2.3 Coupon and incentive management

After completing the questionnaire and receiving posttest counseling, participants were provided with three coupons to recruit their peers. These coupons were valid for up to 2 months and were used to identify the individuals enrolled in the study by a specific participant, allowing researchers to track the reference chain. The process of recruiting peers was repeated in waves until the desired sample size was reached, with a total of 13 waves. The coupon number identified the individuals enrolled in the study by a particular participant and mapped the chain of reference to the researchers.

In order to encourage people participating in the study to include their peers in the study, a primary incentive to participate in the study and a secondary incentive such as money or some other things depending on the existing conditions were provided to them as an incentive. As an incentive for participation, participants received a primary incentive of Rls. 200,000 (approximately US$1.5) upon completing the study and tests. Additionally, they received a secondary incentive of Rls. 300,000 (approximately US$1) for each successful referral of a peer, with a maximum of three peers.

### 2.4 Data collection

Data were gathered on demographics such as age, education level, marital status, and income source and on women's sexual behavior, including age at first sexual contact, the behavioral serological study on FSWs, including information on lifetime and last month alcohol use (self‐report), history of drug use, sexual relations with clients (any sexual activity that FSWs engage in with their clients, which includes vaginal, anal, and oral sex), use of condoms in the last vaginal, anal, or oral sex contact (yes, no), history of group sex (any experience that FSWs may have had with participating in group sex, which involves engaging in sexual activities with multiple partners at the same time), anal sex, and history of intentional abortion (a pregnancy termination that is deliberately sought by the woman, as opposed to a spontaneous abortion (miscarriage) or a medically necessary abortion) was used.

To monitor RDS assumptions, the following activities were carried out: (1) training of the executive team, (2) monitoring and quality control during the data collection stage (observational monitoring, remote monitoring with utilizing the Internet, and monitoring trends), and (3) ensuring and controlling the quality of data entered into the software at the Excel file stage.

In the data collection stage, one or two sampling sites were chosen for each city based on the popular locations and the distance between them. Two individuals served as questioners and samplers at each site, although their number was fewer than the number of seeds due to some seeds being active only on certain days (about 30 people). To adhere to ethical principles, prospective participants were required to go through the process of obtaining informed consent before commencing the study. The participants were asked to sign if they want to participate in the study, and if they do not want to, give their consent verbally.

### 2.5 Data entry and analysis

**FIGURE 1:**
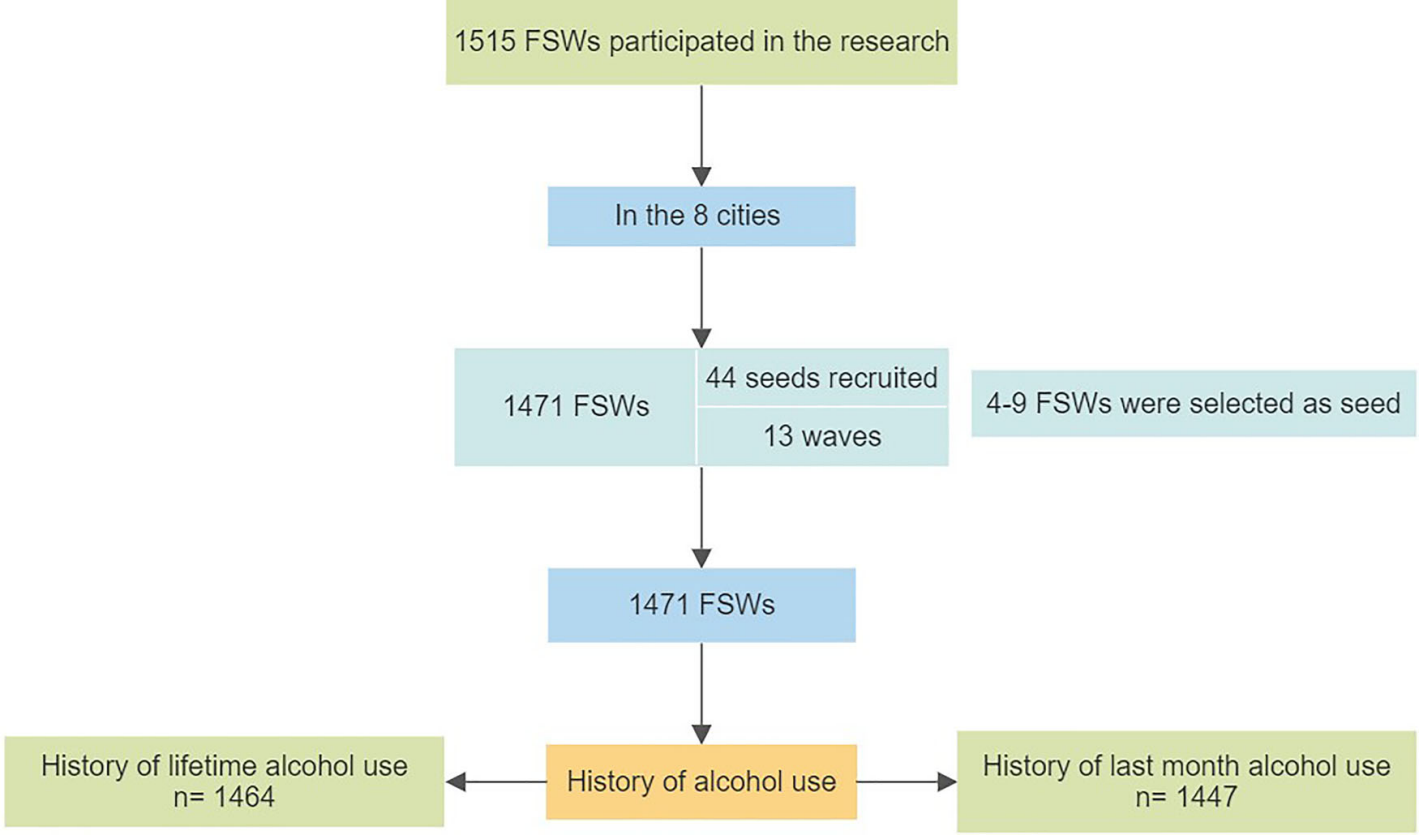
Flow diagram number of participants lifetime and past‐month alcohol use among FSWs in Iran, 2019–2020.

In the present study, after cleaning the data in terms of missing items and the possibility of errors in data entry, data related to all cities were merged. Finally, 1464 FSWs who answered the questions related to alcohol use were included in the analysis, and the rest were not analyzed due to lack of answers. The study began in December 2019 and initially examined approximately 1100 FSWs. However, due to the onset of the COVID‐19 pandemic in March 2020 and subsequent traffic and social restrictions, data collection was interrupted for about 3 months, leading to the suspension of the program. In June 2020, information was collected from the remaining participants (Figure 1).

The primary variables of interest included women's with history of lifetime alcohol use, the number of times of alcohol use in the past‐month as a 4‐level variable (daily, once a week, less than once a week, and nonuse) which in the logistic regression analysis became a binary variable of use and nonuse in the past‐month.

First, descriptive statistics (mean, standard deviation (SD), and percentage) were calculated for sociodemographic and behavioral variables. Bivariate associations between lifetime and in the past‐month alcohol use (as a 4‐level variable, daily, once a week, less than once a week, and nonuse) and these variables were examined using *χ* ^2^ tests for categorical variables or Student's *t* tests for continuous variables. All the variables with a *p*‐value <.05 at the univariable model were included in the multivariable analysis. Univariate and multivariate logistic regression was performed using non‐weighted analysis based on the findings in latest article (Avery et al., 2019). Stata software (version 14), RDS analysis, and R (version 4.1.2) were used to perform all analyses.

### 2.6 Ethical considerations

All procedures performed in the study conformed to the ethical standards of the Kurdistan University of Medical Sciences Committee (ID = IR.MUK.REC.1398.132). The study was anonymous, and all women were informed about the study and asked for written informed consent to participate.

## 3 RESULTS

In this research, 1464 FSWs with a mean age (SD) of 35.66 years (12.32) were studied. The most alcohol used in lifetime and past‐month (weekly, less than once a week, and daily) in FSWs was 52.7% (12.25%, 12.94%, and 1.83%), respectively. A higher frequency of lifetime alcohol use was reported among FSWs who had age group ≤30 years (42.34%), divorced marriage status (42.67%), diploma level of education (29.14%) and have income sources of sex (80.62%). Alcohol use in the last month according to different variables is shown in Table 1. The prevalence of lifetime alcohol use in HIV positive sero‐statue, history of lifetime group sex, and history of lifetime drug use was 1.10%, 23.60%, and 34.36%, respectively. Moreover, the prevalence of lifetime alcohol use in people with the history of intentional abortion was 42.92%, whereas in FSWs who used condoms in the last sexual relation and those who had history of lifetime anal sex was 68.61% and 56.75%, respectively. The prevalence of weekly alcohol use in FSWs with anal sex was higher (Table 2).

**TABLE 1:** Demographic characteristics by the history of lifetime and past‐month alcohol use among FSWs in Iran, 2019–2020.

	History of lifetime alcohol use (*N* = 1464), number^a^ (weighted%)^b^			History of past‐month alcohol use (*N* = 1447), number^a^ (weighted%)^b^				
Variable	**Yes** **900 (52.7)**	**No** **564 (47.3)**	***p*‐Value**	**Daily** 32 (1.83)	**Weekly** 229 (12.25)	**Less than once a week** 265 (12.94)	**No** 921 (72.98)	***p*‐Value**
Age group ≤30 years	340 (42.34)	132 (21.49)	<.001	15 (67.25)	113 (57.46)	99 (40.46)	239 (26.00)	<.001
31–40 years	359 (38.88)	224 (40.52)		12 (26.49)	85 (33.81)	105 (44.32)	375 (40.04)	
41–50 years	173 (16.56)	163 (29.53)		4 (5.36)	26 (7.87)	54 (14.62)	247 (27.12)	
≥51 years	28 (2.23)	45 (8.47)		1 (0.91)	5 (0.86)	7 (0.61)	60 (6.85)	
Marital status single	112 (15.43)	33 (5.18)	<.001	5 (10.72)	34 (19.71)	35 (19.24)	69 (7.42)	<.001
Married	169 (21.68)	144 (28.79)		3 (12.20)	36 (18.70)	46 (18.06)	227(27.76)	
Divorced	419 (46.71)	252 (45.30)		18 (64.72)	105 (45.14)	113 (43.28)	426 (46.25)	
Temporarily married^c^	116 (8.20)	55 (8.15)		4 (4.42)	31 (8.66)	45 (11.18)	89 (7.62)	
Widow	40 (4.31)	71 (11.92)		1 (0.91)	8 (2.45)	11 (3.85)	90 (9.75)	
Living with partner	39 (3.67)	5 (0.66)		1 (7.04)	14 (5.35)	13 (4.39)	15(1.21)	
Level of education illiterate	49 (4.58)	57 (12.02)	<.001	3 (3.80)	10 (1.47)	12 (4.05)	80 (10.07)	<.001
Elementary	156 (17.56)	141 (26.22)		3 (11.45)	38 (18.35)	42 (15.73)	209 (23.54)	
Middle school	203 (24.18)	147 (25.81)		10 (26.62)	43 (21.23)	49 (22.05)	243 (26.07)	
High school	114 (12.07)	59 (9.76)		2 (2.16)	36 (14.76)	32 (10.80)	102 (10.62)	
Diploma	266 (29.14)	114 (18.82)		9 (31.95)	80 (37.00)	86 (27.77)	202 (21.23)	
Academic	111 (12.48)	46 (7.36)		5 (24.02)	22 (7.18)	44 (19.59)	84 (8.46)	
Income source sex	710 (80.62)	428 (79.20)	.582	27 (88.86)	184 (83.56)	218 (86.44)	695 (77.89)	.028
Other	125 (19.38)	105 (20.80)		3 (11.14)	31 (16.44)	28 (13.56)	168 (22.11)	

^a^ Number: crude frequency from sample.

^b^ Weighted: computed by methods: “RDS‐II”.

^c^ Temporary marriage is locally named Sigheh.

**TABLE 2:** Behavioral characteristics, sexual risk factors by history of lifetime, and past‐month alcohol use among FSWs in Iran, 2019–2020.

	History of lifetime alcohol use (*N* = 1464), number^a^ (weighted%)^b^			History of past‐month alcohol use (*N* = 1447) (weighted%)^b^				
Variable	**Yes** 900 (52.7)	**No** 564 (47.3)	***p*‐Value**	**Every day** 32 (1.83)	**Weekly** 229 (12.25)	**Less than once a week** 265 (12.94)	**No** 921 (72.98)	***p*‐Value**
Number	900 (52.7)	564 (47.3)		32 (1.83)	229 (12.25)	265 (12.94)	921 (72.98)	
History of lifetime drug use yes	364 (34.36)	135 (24.42)	<.001	12 (28.66)	81 (31.65)	80 (24.32)	318 (30.31)	.385
No	533 (65.64)	412 (75.58)		20 (71.34)	147 (68.35)	185 (75.68)	585 (69.69)	
Present drug use yes	212 (20.74)	100 (17.82)	.186	6 (15.69)	55 (20.45)	42 (15.15)	203 (20.02)	.438
No	684 (79.26)	447 (82.18)		26 (84.31)	173 (79.55)	223 (84.85)	699 (79.98)	
HIV result negative	889 (98.90)	553 (97.86)	.182	32 (100.0)	226 (99.60)	263 (98.96)	904 (98.07)	.360
Positive	11 (1.10)	11 (2.14)		0 (0.00)	3 (0.40)	2 (1.04)	17 (1.93)	
History of lifetime sex group yes	333 (23.60)	69 (8.22)	<.001	11 (18.51)	106 (32.84)	114 (28.23)	163 (11.22)	<.001
No	550 (76.40)	484 (91.78)		21 (81.49)	121 (67.16)	144 (71.77)	740 (88.78)	
History of intentional abortion yes	395 (42.92)	186 (32.38)	<.001	20 (58.87)	104 (46.67)	116 (39.57)	334 (35.49)	.008
No	425 (57.08)	336 (67.62)		11 (41.13)	105 (53.33)	125 (60.43)	513 (64.51)	
Number of clients (past‐month) 1	385 (63.25)	274 (68.46)	.001	14 (57.37)	93 (56.66)	84 (47.40)	462 (71.28)	<.001
2–5	250 (27.91)	127 (28.75)		11 (32.64)	72 (32.73)	95 (37.58)	193 (25.33)	
≥6	140 (8.85)	20 (2.79)		3 (9.99)	46 (10.62)	54 (15.02)	55 (3.49)	
Using of condom in last sex yes	602 (68.61)	400 (71.49)	.265	19 (68.71)	151 (65.56)	179 (70.15)	643 (70.79)	.577
No	293 (31.39)	161 (28.51)		13 (31.29)	78 (34.44)	85 (29.85)	272 (29.21)	
History of lifetime anal sex yes	584 (56.75)	195 (29.16)	<.001	22 (52.89)	175 (71.67)	182 (52.18)	388 (36.99)	<.001
No	290 (43.25)	355 (70.84)		10 (47.11)	51 (28.33)	76 (47.82)	505 (63.01)	

^a^ Number: crude frequency from sample.

^b^ Weighted: computed by method: “RDS‐II”.

^c^ From total sample.

Based on the results of multivariate logistic regression, several factors were associated with the pattern of lifetime alcohol use among FSWs. The odds ratio (odds) of lifetime alcohol use in FSWs in the age group 31–40 years was 2.41 times higher than ≤30 years (AOR = 2.41, 95% CI: 1.13–5.15). Moreover, the odds of lifetime alcohol use of FSWs in married, temporarily married, and people living with their partners were, respectively, 2.93, 1.88, and 5.04 times higher than single, and there was a significant association with lifetime alcohol use. FSWs with education level of diploma had a higher odds of lifetime alcohol use (AOR = 2.43, 95% CI: 1.31–4.51), and women whose main income source was sex had a higher odds of lifetime alcohol use (AOR = 1.39, 95% CI: 1.05–1.85). FSWs with a history of lifetime drug (AOR = 2.79, 95% CI: 2.01–3.89), history of intentional abortion (AOR = 2.47, 95% CI: 1.82–3.35), having more than six clients in the last month (AOR = 3.25, 95% CI: 1.80–5.87), history of group sex (AOR = 2.07, 95% CI: 1.41–3.03), and history of anal sex in the past‐month (AOR = 2.47, 95% CI: 1.82–3.25) were positively associated with lifetime alcohol use (Table 3).

**TABLE 3:** Factors associated with the history of life time and past‐month alcohol use among FSWs in Iran, 2019–2020.

Variable	Odds ratios (95% confidence intervals)				Odds ratios (95% confidence intervals)			
	**History of lifetime alcohol use (*N* = 1464)**				**History of last month alcohol use (*N* = 1447)**			
	OR (95%CI)	*p*‐Value	AOR (95%CI)	*p*‐Value	OR (95%CI)	*p*‐Value	AOR (95%CI)	*p*‐Value
Age group ≤30 years	Ref.	–	Ref.	–	Ref.	–	Ref.	–
31–40 years	4.14 (2.48, 6.91)	<.001	2.41 (1.13, 5.15)	.023	4.38(2.34, 8.20)	<.001	2.61(1.11, 6.14)	.028
41–50 years	2.58 (1.56, 4.25)	<.001	1.44 (.69, 2.98)	.331	2.49 (1.33, 4.64)	.004	1.57 (.68, 3.64)	.292
≥51 years	1.71 (1.02, 2.86)	.043	1.23 (.59, 2.58)	.578	1.57 (.82, 3.00)	.173	1.13 (.48, 2.68)	.774
Marital status single	Ref.	–	Ref.	–	Ref.	–	Ref.	–
Married	6.02 (3.48, 10.43)	<.001	2.93 (1.36, 6.33)	.006	4.83 (2.69, 8.66)	<.001	2.33 (1.06, 5.15)	.036
Divorced	2.08 (1.33, 3.26)	.001	1.43 (.79, 2.60)	.242	1.69 (.98, 2.91)	.061	.99 (.49, 2.00)	.973
Temporarily married	2.95 (1.94, 4.48)	<.001	1.88 (1.06, 3.35)	.032	2.49 (1.50, 4.15)	<.001	1.42 (.73, 2.78)	.304
Widow	3.74 (2.26, 6.19)	<.001	1.86 (.96, 3.62)	.067	4.04 (2.29, 7.16)	<.001	1.81 (.87, 3.77)	.112
Living with partner	13.85 (5.05,37.95)	<.001	5.04 (1.01, 25.05)	.048	8.4 (3.80, 18.55)	<.001	2.25 (.71, 7.17)	.169
Level of Education Illiterate	Ref.	–	Ref.	–	Ref.	–	Ref.	–
Elementary	1.29 (.83, 2.01)	.266	1.52 (.83, 2.79)	.176	1.27 (.76, 2.13)	.363	1.37 (.70, 2.71)	.359
Middle school	1.61 (1.04, 2.49)	.033	1.19 (.65, 2.16)	.577	1.34 (.81, 2.23)	.252	1.04 (.53, 2.03)	.908
High school	2.25 (1.37, 3.69)	.001	1.15 (.58, 2.28)	.679	2.20 (1.28, 3.78)	.004	1.30 (.63, 2.70)	.482
Diploma	2.71 (1.75, 4.22)	<.001	2.43 (1.31, 4.51)	.005	2.77 (1.69, 4.54)	<.001	2.06 (1.06, 4.01)	.032
Academic	2.81 (1.68, 4.69)	<.001	1.50 (.71, 3.18)	.284	2.70 (1.56, 4.68)	<.001	1.40 (.65, 3.01)	.391
Income source other	Ref.	–	–	–	Ref.	–	Ref.	–
Sex	1.39 (1.05, 1.85)	.023	–	–	1.67 (1.22, 2.29)	.001	1.60 (1.09, 2.34)	.016
History of lifetime drug use no	Ref.	–	Ref.	–	Ref.	–	–	–
Yes	2.08 (1.65, 2.64)	<.001	2.79 (2.01, 3.89)	<.001	.90 (.72, 1.13)	.385	–	–
History of lifetime group sex no	Ref.	–	Ref.	–	Ref.	–	Ref.	–
Yes	4.25 (3.19, 5.66)	<.001	2.07 (1.41, 3.03)	.001	3.67 (2.88, 4.67)	<.001	1.81 (1.29, 2.54)	.001
History of intentional abortion no	Ref.	–	Ref.	–	Ref.	–	Ref.	–
Yes	1.68 (1.34, 2.10)	<.001	1.42 (1.06, 1.92)	.021	1.53 (1.22, 1.92)	<.001	1.42 (1.06, 1.91)	.019
Number of clients (past‐month) 1	Ref.	–	Ref.	–	Ref.	–	Ref.	–
2–5	1.40 (1.08, 1.82)	.012	1.13 (.82, 1.56)	.449	2.23 (1.71, 2.91)	<.001	1.82 (1.32, 2.50)	<.001
≥6	4.98 (3.04, 8.16)	<.001	3.25 (1.80, 5.87)	<.001	4.53 (3.13, 6.55)	<.001	2.81 (1.78, 4.44)	<.001
History of lifetime anal sex no	Ref.	–	Ref.	–	Ref.	–	Ref.	–
Yes	3.67 (2.93, 4.59)	<.001	2.47 (1.82, 3.35)	<.001	3.60 (2.84, 4.56)	<.001	2.11 (1.52, 2.93)	<.001

Based on the results of multivariate logistic regression, several factors were related to the pattern of alcohol use in the recent month among FSWs. Alcohol use in the past‐month was significantly higher in FSWs age group 31–40 years (AOR = 2.61, 95% CI: 1.11–6.14), married FSWs (AOR = 2.33, 95% CI: 1.06–5.15), education level of diploma (AOR = 2.06, 95% CI: 1.06–4.01), main source of income was sex (AOR = 1.60, 95% CI: 1.09–2.33), history of intentional abortion (AOR = 1.42, 95% CI: 1.06–1.91), having more than six clients in the past‐month showed a (AOR = 2.81, 95% CI: 1.78–4.44), history of sex group (AOR = 1.81, 95% CI: 1.29–2.54), and FSSWs who reported a history of anal sex were positively associated with past‐month alcohol use (AOR = 2.11, 95% CI: 1.52–2.93) (Table 3).

## 4 DISCUSSION

The results showed that the prevalence of lifetime and past‐month (weekly, less than once a week, and daily) alcohol use in FSWs was 52.7% (12.25%, 12.94%, and 1.83%), respectively. The global prevalence of alcohol use disorders among women in the general population is 5.1% (Degenhardt et al., 2018). The results of a meta‐analysis in Iran showed that the prevalence of lifetime alcohol use in the whole population among women and young women was 7% and 8%, respectively (Chegeni et al., 2020). Moreover, the prevalence of alcohol use among FSWs in different studies in Iran was about 52% (Karamouzian et al., 2017; Mousavi‐Ramezanzade et al., 2020).

The results of a meta‐analysis alcohol use among FSWs in low‐ and middle‐income countries showed for studies reporting on alcohol use frequency, 12%–100% used alcohol in the past‐month; 89%–93.7% reported using alcohol in the last 12 months; 6.4%–77.8.0% used alcohol at least once a week and 10%–64.6% used alcohol at least once a month (Beksinska et al., 2023). A study conducted in Brazil showed that the consumption of alcohol and other beverages in FSWs was approximately 85% (Fernandes et al., 2014), whereas in Kenya, it was about 30% (Chersich et al., 2007a).

All these data indicate a significantly higher burden of harmful alcohol use among FSWs. Alcohol use is prevalent during sex work and on entry into sex work, which reflects previous evidence about the availability and normalization of alcohol in the sex work industry (Li et al., 2010; Mbonye et al., 2014).

The high burden of alcohol use has serious health and social implications for FSWs, as excess alcohol use is associated with multiple poor physical and mental health outcomes (Rehm, 2011). Based on the results observed among FSWs, necessary interventions are essential to reduce alcohol use in this group (Chersich et al., 2007a). In general, our research highlights the need for prevention and harm reduction measures regarding alcohol use among FSWs, taking into account the proportion and resulting consequences. It is essential to conduct further studies to assess the effectiveness of interventions aimed at reducing alcohol‐related harm, such as altering drinking behaviors and minimizing potentially risky sexual practices. Brief interventions have proven to be remarkably successful in various contexts and could be particularly suitable for this purpose (Organization, 2001).

The results of the present study showed that age group 31–40 years in FSWs was associated with lifetime and in the past‐month alcohol use. Young people are more likely to engage in undesirable behaviors than older ones in the society (Jorjoran Shushtari et al., 2021). In fact, less alcohol use in Iran can be rooted in its sociocultural and religious context. However, evidence suggests that alcohol use among young people in Iran may have increased over the last few decades. This indicates that young FSWs should be a target for alcohol interventions to prevent long‐term alcohol related harms (Bryden et al., 2012; Walls et al., 2020).

We found that married, temporarily married, and ones living with partner status were associated with increased likelihood of lifetime alcohol use in FSWs. As the results of other studies showed a decrease in alcohol use in married people (Martin, 2019; Reczek et al., 2014) although the protective effect of marriage in women is less in some studies and more in others (Martin, 2019; Reczek et al., 2016). The history of family alcohol use, access to alcohol, and living with a partner who alcohol use may contribute in use or non‐alcohol among FSWs.

The results of the present study showed that lifetime alcohol use among FSWs had a significant association with the education level of diploma. The findings of other studies indicated that alcohol use and other high‐risk behaviors were higher among people with lower education (Hingson et al., 2017; Lorant et al., 2013; Quinn & Fromme, 2011). The results of studies in China also showed that alcohol use was higher in FSWs with higher education (Chen et al., 2012; Lui et al., 2018). In another study, it was found that there was no association between the level of education and alcohol use. These results indicate a contradiction among different studies (Wang et al., 2010). Therefore, it is crucial to conduct further research to explore the association between education level and alcohol use among FSWs.

In a cross‐sectional study involving FSWs, it was found that sex work constituted the primary source of income for 97% of the women (Kakisingi et al., 2020). Another study conducted by Roshanfekr et al. (2015) revealed that approximately half of the women engaged in sex work to cover expenses resulting from financial difficulties, making it their main source of income. This finding is consistent with the results of the present study.

One of the factors affecting alcohol use in this study was history of group sex among FSWs. Although a study in Melbourne, Australia, did not show an association between group sex and alcohol use, but unsafe sex group (not using a condom) was associated with an increased risk of HIV and STIs, whereas the risk of alcohol and drug use was higher among several sexual partners in a shared risky environment (Turek et al., 2020; Violette et al., 2019).

The results of the present study showed that FSWs with a history of intentional abortion had higher odds of alcohol use. A survey on Iranian FSWs in 2010 reported that about 35% of FSWs had a lifetime abortion history (Turek et al., 2020). One explanation for this finding is the social stigma and negative attitudes of the society toward FSWs, which make access to contraceptive services more difficult. The lack of financial and family support for the future baby increases the decision to have an abortion (Shokoohi et al., 2019).

Results of studies have found a positively associated between the number of clients an FSWs has and her alcohol use which is consistent with our study. The FSWs who have a larger number of clients may be exposed to more stressful and traumatic experiences, which can lead to alcohol use as a coping mechanism (Quinn & Fromme, 2011; Zhang et al., 2013).

Based on this study, financial problems or overpayments for anal sex were the main reasons why FSWs were involved in anal sex. Making more money is a common reason for many FSWs to have anal sex, as shown in other studies (Chersich et al., 2007b; Patra et al., 2012).

The limitations of this study included the following. Due to the illegal and unconventional nature of sex worker in most countries, including Iran, sampling and follow‐up were difficult, especially since the sampling period of this study coincided with the COVID‐19 pandemic and led to an interruption in the data collection process. To reduce the effects of this interruption, the researchers only included women who had a coupon from a previous participant. It was also a cross‐sectional study and like other cross‐sectional ones did not allow the researcher to examine the causal association between the outcome variable and the independent variables. Although the RDS sampling method has been used in this study, which is a good method for sampling in marginal groups, the results of this research should be generalized to all groups with caution due to the lack of coverage of all different groups of FSWs. One of the main limitations in the field of FSWs is that a consent letter for FSWs under 18 years of age must be obtained from the legal guardian of these people, whereas most of these people do not have a guardian or cannot be reached at all. This issue leads to this important group being ignored. Despite the limitations, the findings of this study can be used to monitor this high‐risk behavior in this group over time.

## 5 CONCLUSION

Alcohol use is prevalent among FSWs in Iran. The results of this study by determining some indicators related to alcohol use among FSWs can help to observe its changes over time and improve the monitoring system of this behavior. Further prevention programs are needed to address and reduce harms associated with alcohol use among this vulnerable population in Iran. Designing intervention programs, it is suggested to consider other variables affecting alcohol use in FSWs.

## AUTHOR CONTRIBUTIONS

Ghobad Moradi, Mohammad Mehdi Gouya, Mohammad Aziz Rasouli, and Bushra Zareie conceived and designed the study. Bushra Zareie, Mohammad Aziz Rasouli, and Ghobad Moradi analyzed and interpreted the data and drafted the manuscript. Mohammad Aziz Rasouli, Ghobad Moradi, Bushra Zareie, Fatemeh Hadavandsiri, Marzieh Mahboobi, Yousef Moradi, Mohammad Mehdi Gouya, and Rozhin Moradi were involved in the composition of the study tool, collect data, supervision of the research process, and critical revision and review of the manuscript. All the authors read and approved the final manuscript.

## CONFLICT OF INTEREST STATEMENT

The authors declare that they have no conflicts of interest.

### PEER REVIEW

The peer review history for this article is available at https://publons.com/publon/10.1002/brb3.3288.

## Data Availability

The data that support the findings of this study are available from the corresponding author upon reasonable request.
